# Zoonotic endoparasites and *Toxoplasma gondii* seropositivity in free-roaming cats (*Felis catus*) from New York City boroughs

**DOI:** 10.1371/journal.pone.0351437

**Published:** 2026-07-08

**Authors:** Viet-Linh Nguyen, Elizabeth Gurtowski, Jiayi Chen, Megan Rosen, Pratap Kafle

**Affiliations:** 1 Shreiber School of Veterinary Medicine, Rowan University, Harrison Township, New Jersey, United States of America; 2 Lewyt College of Veterinary Medicine, Long Island University, Brookville, New York, United States of America; Guru Angad Dev Veterinary and Animal Sciences University, INDIA

## Abstract

Free-roaming cats (*Felis catus*) can serve as reservoirs of various zoonotic parasites in urban settings. Despite a large population of free-roaming cats around New York City, studies assessing the prevalence and shedding of various parasites in the New York urban landscape are scarce. This study utilized fecal and blood samples opportunistically collected during the Trap Neuter Return (TNR) program from 87 free-roaming cats in New York City between May and July 2023. Samples were analyzed using centrifugal fecal flotation, coproantigen immunoassays, serologic assays, and PCR-based assays for gastrointestinal and vector-borne parasites. Fecal flotation (n = 87) results revealed that 57.5% (50/87; 95% CI: 46.9–67.4) of cats were infected with at least one species of parasite. The most prevalent infection was *Toxocara* spp. (54%; 95% CI: 43.4–64.3), followed by *Ancylostoma* spp. (13.8%; 95% CI: 8.2–22.6) and coccidia (11.5%; 95% CI: 6.4–19.9). Coproantigen testing (n = 43) identified *Giardia* spp. in 11.6% (5/43; 95% CI: 5.1–24.5) and *Cryptosporidium* spp. in 2.3% (1/43; 95% CI: 0.4–12.1) of cats. Antibodies to *Toxoplasma gondii* were detected in 8.9% (4/45; 95% CI: 3.5–20.7) of serum samples; no *Dirofilaria immitis* antigen and *Cytauxzoon felis* DNA were found in the blood samples (n = 45). Male cats were significantly more likely to be infected with *Toxocara* spp. (OR = 4.36) and, along with juvenile cats (<1 year), shed significantly higher numbers of eggs (p < 0.05), identifying young males as high-intensity “super-shedders” driving environmental contamination. The high prevalence of zoonotic helminths, particularly *Toxocara* spp., underscores the public health risks associated with unmanaged feline populations in densely populated urban centers. These findings highlight the utility of integrating disease surveillance into TNR programs to monitor urban ecosystem health and mitigate zoonotic risks.

## Introduction

Free-roaming cats (*Felis catus*), including stray, feral, and outdoor-access owned cats, are abundant in many densely populated urban environments, where they occupy shared spaces with people, pets, and synanthropic wildlife, increasing the likelihood of environmental exposure to various parasites and pathogens through contaminated soil and public areas. These cats can serve as significant reservoirs for a range of helminths and protozoal parasites, such as *Toxocara* spp., *Ancylostoma* spp., *Giardia* spp., *Cryptosporidium* spp., and *Toxoplasma gondii*, that can cause diseases in companion animals and humans, with children and immunocompromised individuals at particular risk [1–5]. From a One Health perspective, unmanaged free-roaming cat populations occupy a critical interface between animal health, human health, and environmental hygiene. Therefore, surveillance of parasitic infections in urban free-roaming cat populations represents an important component of One Health approaches aimed at understanding and mitigating zoonotic risks at the human–animal–environment interface.

Environmental persistence of parasite stages shed by cats further amplifies their public health significance. For instance, eggs of *Toxocara* spp. are highly resistant to environmental degradation and can remain infective in soil for prolonged periods, leading to widespread contamination of public spaces, indicating potential exposure for people and other animals [6]. Similarly, *T. gondii* oocysts shed by infected cats can persist in soil and water, contaminate produce, and infect terrestrial and aquatic wildlife, illustrating the cross-ecosystem nature of this parasite [7–9]. *Giardia* and *Cryptosporidium* infections in cats involve a mixture of host-adapted and potentially zoonotic assemblages and species, and while the overall zoonotic risk from cats is considered low to moderate, their role as sources and sentinels remains relevant in densely populated settings [3].

In many cities, Trap Neuter Return (TNR) programs are used to manage feral cat colonies, and they offer an efficient opportunity to integrate disease surveillance into ongoing population control and welfare interventions. Leveraging TNR programs for systematic parasitological monitoring can generate data that are directly relevant to One Health, including estimates of infection prevalence, intensity of environmental shedding, and demographic risk factors within urban cat populations.

The objectives of this study were to estimate the prevalence of key gastrointestinal and zoonotic parasites in free-roaming cats enrolled in a TNR program in NYC and to identify demographic risk factors associated with infection and shedding intensity. These data provide an important baseline for understanding the role of free-roaming cats in environmental contamination and inform One Health strategies to mitigate zoonotic risks in densely populated urban environments.

## Materials and methods

### Study population and sampling

**Fig 1:**
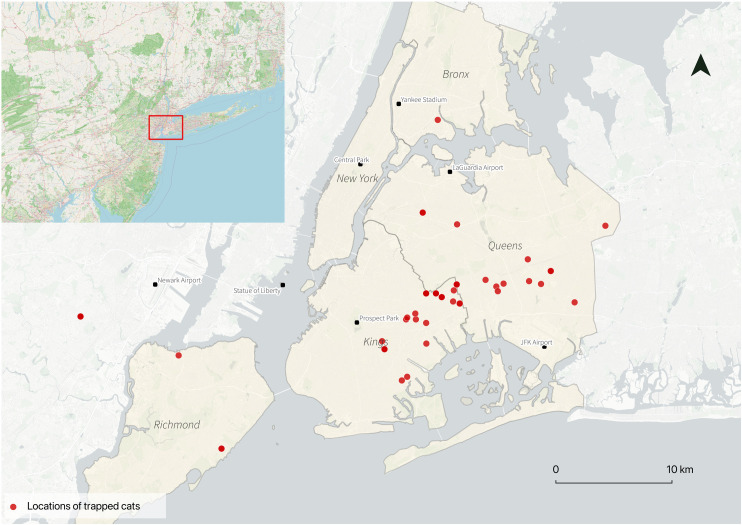
Sampling locations of free-roaming cats trapped through the Long Island University College of Veterinary Medicine Trap Neuter Return program, New York City, USA, May–July 2023 (n = 87). Red points denote trap-site coordinates rounded to two decimal places (~1.1 km grid). The figure was prepared in QGIS 3.34 using public-domain shapefiles.

This cross-sectional study was conducted between May and July 2023 using free-roaming cats (defined here as cats not confined indoors and including feral, stray, and colony-associated individuals) captured through the Long Island University College of Veterinary Medicine TNR program from various locations around NYC (Fig 1). Cats were humanely trapped at multiple sites, and the trapping location was recorded. For each individual cat, the attending veterinarians recorded sex and reproductive status, including pregnancy and lactation. Age estimation was based on physical examination, dentition, and body size.

Following physical examination, each cat received a unique microchip identifier and was surgically sterilized as part of the TNR program. The animals were then released at the site of capture. The study procedures complied with institutional guidelines for animal handling and welfare and were approved by the Institutional Animal Care and Use Committee of Long Island University (protocol # 2023−021).

### Laboratory analyses

Fresh fecal samples were collected rectally and processed within 24 hours. Quantitative fecal flotation was performed using the Wisconsin double-centrifugation technique with Sheather’s sugar solution (Jorgensen Laboratories, CO; specific gravity 1.27). Briefly, a precisely weighed fecal sample (~2 g, recorded to 0.01 g) was suspended in tap water, strained through cheesecloth, and centrifuged at 400 × g for 5 minutes to pellet the eggs. The supernatant was decanted, the pellet was resuspended in Sheather’s solution, and the suspension was centrifuged a second time at 400 × g for 5 minutes with a coverslip placed on top of a meniscus formed at the tube rim. The coverslip was transferred to a microscope slide, and the entire coverslip area was systematically examined at 100× and 400 × magnification. Helminth eggs and protozoal oocysts were identified based on morphological characteristics [10]. For each sample, all eggs and oocysts on the entire coverslip were enumerated, and eggs per gram (EPG) were calculated by dividing the total egg count by the recorded weight of the fecal sample in grams. This method is widely accepted as a quantitative fecal test yielding EPG values with sensitivity advantages for low- and moderate-intensity infections [11,12]. Due to limited fecal sample volumes, traditional zinc sulfate (ZnSO4) centrifugal flotation for *Giardia* cyst detection was omitted; instead, samples were processed for coproantigen testing, which is considered more sensitive for detecting *Giardia* antigen. Also, the insufficient fecal material precluded the use of fecal sedimentation and the Baermann technique, restricting the parasitological assessment to flotation and antigen-based diagnostics only.

### Antigen-based, serologic and molecular testing

Due to sample volume and assay availability, coproantigen and serologic testing were performed on subsets of available samples. For this, fecal, serum, and whole blood samples were submitted to the Cornell University Animal Health Diagnostic Center (AHDC) for further analysis.

Fecal samples (n = 43) were tested for *Giardia* and *Cryptosporidium* using ProSpecT™ Giardia Microplate Assay (Thermo Fisher, USA) and ProSpecT™ Cryptosporidium Microplate Assay (Thermo Fisher, USA), respectively. Although these commercial enzyme-linked immunosorbent assays (ELISA) were originally developed for human diagnostics, they have been validated for veterinary species at the Cornell AHDC and are verified in-house for the detection of *Giardia* and *Cryptosporidium* in veterinary samples. These assays are widely used for detection of protozoal infections in animals and contaminated environments with higher sensitivity compared with flotation alone [13]. Antigen-positive samples were further submitted to the Cornell AHDC for molecular confirmation using their validated in-house PCR assays.

Serum samples (n = 45) were screened for *T. gondii* antibodies using the modified agglutination test (MAT), with titers ≥1:25 considered evidence of prior exposure, consistent with previous studies in cats and wildlife [14,15]. A commercial heartworm antigen ELISA was performed by the Cornell AHDC as part of their standard diagnostic service to detect circulating *Dirofilaria immitis* adult-female antigen.

EDTA whole-blood samples (n = 45) were submitted to the Cornell AHDC for detection of *Cytauxzoon felis* using their validated in-house real-time PCR diagnostic assay.

### Data analysis

Parasite prevalence was calculated separately for each diagnostic method using appropriate denominators: fecal flotation (n = 87), protozoal antigen testing (n = 43), and *T. gondii* serology (n = 45). Prevalence estimates were reported as percentages with 95% confidence intervals. Associations between host demographic factors such as age (young cats: < 1 year old, comprising kittens and juveniles; adults: ≥ 1 year old), sex, lactation status and parasite infection status were assessed using chi-square tests or Fisher’s exact tests when expected cell counts were less than 5, with statistical significance evaluated at p ≤ 0.05. Odds ratios (OR) with 95% confidence intervals were calculated for significant predictors.

Multivariable logistic regression models were constructed to assess independent effects of age and sex on binary infection outcomes (any parasite infection) while controlling for potential confounding. Model coefficients were exponentiated to yield adjusted odds ratios (aOR) with 95% confidence intervals.

For infection intensity analysis, overdispersion in egg per gram (EPG) count data was assessed by calculating variance-to-mean ratios. Due to substantial overdispersion (variance/mean ratio = 835.7), negative binomial generalized linear models (GLMs) were fitted to model EPG as a function of age and sex. Incidence rate ratios (IRR) with 95% confidence intervals were calculated to quantify the magnitude of associations. Non-parametric Mann-Whitney U tests were used to compare EPG distributions between two groups, and Kruskal-Wallis tests were employed for comparisons across three age categories (kittens, juveniles, and adults). All statistical analyses were performed using R version 4.3.1 [16] with packages including tidyverse, MASS, binom, epitools, and DescTools.

## Results

### Study population

A total of 87 free-roaming cats were sampled and included in parasitological analysis. Among cats with complete demographic data (n = 59), most were classified as young (<1 year old, n = 43, 72.9%), comprising 5 kittens (<6 months) and 38 juveniles (6–12 months), with 16 adults (>12 months, 27.1%). The population included 19 males and 40 females. Among females with known reproductive status, 11 were pregnant and 7 were lactating at the time of examination.

### Parasitological findings

Overall, 57.5% (50/87; 95% CI: 46.9–67.4%) of cats were positive for at least one endoparasite species detected by fecal flotation. *Toxocara* spp. eggs were the most prevalent, detected in 54.0% (47/87; 95% CI: 43.6–64.1), followed by *Ancylostoma* spp. eggs in 13.8% (12/87; 95% CI: 8.1–22.6), and coccidia oocysts in 11.5% (10/87; 95% CI: 6.4–19.9) (Table 1).

**Table 1:** Prevalence of parasites in free-roaming cats in New York City. Prevalence with 95% Confidence Interval (CI) was calculated using total samples tested per method.

Parasites	Diagnostic method	Positive/ Total	Prevalence (%)	95% CI
**Helminths**				
*Toxocara* spp.	Fecal flotation	47/ 87	54.0	43.6–64.1
*Ancylostoma* spp.	Fecal flotation	12/ 87	13.8	8.1–22.6
**Protozoa**				
Coccidia	Fecal flotation	10/ 87	11.5	6.4–19.9
*Giardia* spp.	Coproantigen ELISA	5/ 43	11.6	5.1–24.5
*Cryptosporidium* spp.	Coproantigen ELISA	1/ 43	2.3	0.4–12.1
*Toxoplasma gondii*	Serology (MAT)	4/ 45	8.9	3.5–20.7

Co-infections were frequently observed, with 21.8% (19/87; 95% CI: 14.5–31.6%) of the cats harboring two or more parasite species simultaneously. The most common combination was *Toxocara* spp. and *Ancylostoma* spp., which was identified in 11.5% (10/87; 95% CI: 6.4–19.9%) of the screened cats.

Antigen testing of 43 fecal samples identified *Giardia* in 11.6% (5/43; 95% CI: 5.1–24.5) and *Cryptosporidium* in 2.3% (1/43; 95% CI: 0.4–12.1), including infections that were not detected by flotation alone. Confirmatory PCR performed by the Cornell AHDC did not detect *Giardia* or *Cryptosporidium* DNA in any sample.

Of the 45 serum samples tested, 8.9% (4/45; 95% CI: 3.5–20.7) were seropositive for *T. gondii* antibodies, indicating prior exposure despite the absence of detectable *T. gondii* oocysts in fecal flotation. No heartworm antigen was detected in any cat, and real-time PCR did not identify *C.* *felis* DNA in any tested blood sample.

### Risk factor analysis

*Risk factor for infection:* Univariable analysis revealed no significant demographic risk factors for the overall presence of parasites. The prevalence of “any parasite” did not differ significantly between young cats (69.8%) and adults (62.5%) (p = 0.60), nor between males (84.2%) and females (60.0%) (p = 0.06). When analyzing specific parasites, male sex was identified as a significant risk factor for *Toxocara* spp. infection (OR=4.36; 95% CI: 1.10–17.37; p = 0.04). No significant demographic associations were found for *Ancylostoma* spp. or coccidia. While lactating females showed a higher prevalence of coccidia (27.3%) compared to non-lactating females (3.4%), this difference approached but did not reach statistical significance (OR=10.5; p = 0.056).

*Infection intensity:* While the prevalence of infection was largely consistent across demographics, the intensity of egg shedding exhibited distinct biological patterns. *Toxocara* spp. egg counts were highly overdispersed and significantly associated with both age and sex (Table 2). A negative binomial GLM identified young cats as “super-shedders,” with an incidence rate ratio (IRR) of 8.74 (p = 0.002) compared to adults. Males also shed significantly higher numbers of *Toxocara* eggs than females (IRR = 3.67; p = 0.030). In contrast, shedding intensity for *Ancylostoma* spp. did not differ significantly by age (Young median: 25.0 EPG vs. Adult median: 94.2 EPG; p = 0.52) or sex (p = 0.69). Similarly, coccidia oocyst counts showed no significant intensity differences between demographic groups (p = 0.29).

**Table 2:** Associations between host demographic factors and both infection prevalence (risk) and egg shedding intensity (burden) for dominant helminths in free-roaming cats around New York City.

Demographic Group	N	*Toxocara* spp.		*Ancylostoma* spp.	
		Prevalence (%) (OR; 95% CI)	Median EPG (IRR; p-value)	Prevalence (%) (OR; 95% CI)	Median EPG (p-value)
**Age**					
**Young (<1 yr)**	43	**67.4** (1.6; 0.5–5.2)	84.2 **(8.74; 0.002)**	16.3 (0.8; 0.2–3.8)	25.0 (0.52)
**Adult (≥1 yr)**	16	56.2 (Reference)	10 (Reference)	18.8 (Reference)	94.2
**Sex**					
**Male**	19	**84.2** **(4.4; 1.1–17.4)**	**109.3** **(3.67; 0.030)**	26.3 (2.5; 0.6–10.0)	247.0 (0.69)
**Female**	40	55.0 (Reference)	10 (Reference)	11.6 (Reference)	25.0

**Note:** EPG = Eggs Per Gram of feces. OR = odds ratio from Fisher’s exact test. IRR = incidence rate ratio from negative binomial generalized linear model. Median egg counts were calculated among infected animals only. **Significant associations are shown in bold.**

## Discussion

This study reports a high burden of gastrointestinal parasites in free-roaming cats around NYC and highlights important demographic patterns with direct relevance to zoonotic risk and environmental contamination in urban environments. More than half of the cats examined were infected with at least one endoparasite species, with *Toxocara* spp. and *Ancylostoma* spp. occurring at prevalences comparable to or exceeding those reported in other urban cat populations in the northeastern United States [17,18]. These findings reinforce the role of free-roaming cats as reservoirs of environmentally persistent zoonotic helminths in densely populated landscapes.

The high prevalence of *Toxocara* spp. found in this population is consistent with the parasite’s ubiquity and ability to contaminate the environment, particularly in urban areas where feral cat colonies exist and feces are not promptly removed. This rate exceeds those reported in many other urban centers in developed nations and aligns more closely with prevalences found in developing regions or rural environments [19]. Recent environmental surveillance by Tyungu et al. (2020) detected *Toxocara* eggs in 38.5% of NYC public playgrounds, with contamination rates reaching 66.7% in the Bronx [17]. Critically, that study identified *T. cati* as the predominant species in soil samples, rather than the canine species *T. canis*. Our findings support the idea that the free-roaming cat population is the active biological source of this environmental burden. Because *Toxocara* *eggs are highly robust, remaining infective in soil for years and*withstanding the northeastern winter freeze-thaw cycles, environmental shedding by these cats presents a long-term public health risk.

The public health implications of these findings are particularly salient in a context such as New York City. Despite NYC’s high Human Development Index and substantial healthcare infrastructure, seroprevalence estimates indicate that approximately 5% of the U.S. population carries antibodies to *Toxocara*, with markedly higher rates among children, non-Hispanic Black populations, and residents of low-income urban neighborhoods [5]. Toxocariasis is recognized by the U.S. Centers for Disease Control and Prevention as one of five neglected parasitic infections of public health concern. Children are at disproportionate risk because of geophagia, hand-to-mouth behavior, and play in soil and sand contaminated by free-roaming-cat feces, exposures that are concentrated in dense urban centers where outdoor space is shared between feral colonies and the public. The substantial playground-soil contamination dominated by *T. cati* that has been documented in NYC public spaces [17], together with the high *Toxocara* prevalence and the heavy juvenile-male shedding intensity reported here, suggests transmission risk. These findings support the idea of integrating environmental decontamination, public education, and targeted parasite control into TNR programs as a One Health priority.

By quantifying egg counts alongside prevalence, this study adds an important dimension to understanding transmission risk: infection intensity is highly aggregated, with a subset of cats responsible for a disproportionate share of environmental egg output. Egg shedding varied greatly by age and sex, even though parasites were present in high numbers across all demographic groups. Young cats were classified as “super-shedders,” demonstrating an egg shedding intensity nearly tenfold greater than that of adults (IRR = 8.74; p = 0.002). This pattern is consistent with age-acquired immunity and reduced worm fecundity in older hosts, as described for ascarid infections in both domestic and wild carnivores [10]. Male cats were identified as being four times more likely to be infected with *Toxocara* spp. (OR=4.36) and showed significantly higher egg-shedding intensity than females (IRR = 3.67; p = 0.030). This male bias has been observed in other mammalian host-parasite systems [20] and is often attributed to the immunosuppressive effects of testosterone or behavioral factors such as larger home ranges that increase exposure to contaminated soil and paratenic hosts [21,22]. From a management standpoint, the emergence of young males as “super-shedders” suggests that targeted interventions in this demographic group could yield outsized reductions in environmental contamination. Administering broad-spectrum anthelmintics to heavily shedding juveniles and young males during TNR procedures may be more efficient, in terms of eggs removed from the environment per treatment, than uniformly treating all cats with low-intensity infections. Operationalizing such targeted deworming will require further work on feasibility, cost, and potential for repeated treatment in colonies, but the principle of focusing on high-shedding individuals is well aligned with modern parasite control strategies. It should be noted, however, that egg-output measures are best interpreted as indices of environmental contamination potential rather than as direct estimates of adult-worm burden, since per-female fecundity in *Toxocara cati* can exceed 100,000 eggs per day and is modulated by immune status, worm age, and density-dependent effects [23].

The observed prevalence of *Ancylostoma* spp. in this population has important implications for both animal and human health in New York City. Although *A. tubaeforme* is the most common feline hookworm, its eggs are morphologically indistinguishable from those of the zoonotic species *A. braziliense*, which cats can also shed [24]. Without molecular speciation, the precise zoonotic risk associated with these infections cannot be determined; however, the presence of a sizeable hookworm-infected free-roaming cat population in a temperate urban setting is noteworthy given ongoing climate-driven shifts in helminth distributions. Reports of *A. braziliense* in dogs across a broad swath of the United States, including northern regions, suggest that ecological conditions are becoming increasingly permissive for subtropical hookworms, warranting continued surveillance and molecular characterization of feline hookworms in the Northeast [25,26]. Coccidia oocysts were also detected at a moderate prevalence. Although species-level identification was not pursued, these oocysts are most likely members of the *Cystoisospora* complex that commonly infects cats and primarily affects young or immunocompromised individuals. While feline coccidia are generally regarded as having limited direct zoonotic relevance, their presence in free-roaming cats is epidemiologically relevant because they can contribute to gastrointestinal disease and poor body condition in kittens. The trend toward higher coccidia prevalence in lactating females further suggests that reproductive and nutritional stress may influence susceptibility or shedding. Together, the hookworm and coccidia findings highlight the need to consider both zoonotic risk and animal welfare when designing parasite control strategies for urban TNR programs.

*Giardia* and *Cryptosporidium* infections in this study were detected using coproantigen assays applied to a subset of fecal samples. In field-based studies of small mesocarnivores, particularly when fecal samples are collected rectally during brief handling windows, available sample volume is severely limited, and degradation cannot always be prevented before processing. Under these constraints, coproantigen ELISAs offer practical advantages: they require minimal fecal material, are more sensitive than flotation for detecting low-intensity or intermittent shedding (especially for *Giardia*), and tolerate suboptimal sample preservation better than microscopy. Conventional flotation, by contrast, requires larger volumes and yields lower sensitivity when oocyst/cyst output is sparse. However, coproantigen assays cannot differentiate zoonotic from host-adapted assemblages, and the absence of PCR amplification likely reflects low parasite loads, intermittent shedding, or inhibitors in field-collected samples rather than true absence. These findings underscore the utility of antigen-based methods for TNR surveillance but highlight the need for future work coupling sensitive antigen detection with genotyping to clarify the specific role of urban cats in *Giardia* and *Cryptosporidium* transmission cycles relevant to human health.

The non-detection of *C. felis* DNA in any cat tested is also noteworthy, given the rapid range expansion of its primary vector, the lone star tick (*Amblyomma americanum*), into New York State [27]. Populations of *A. americanum* are now well-established on Long Island and Staten Island and have been detected in the Bronx [28]. The presence of the competent vector without the pathogen suggests that NYC is currently a “receptive” zone for *C. felis*. The lack of infection in our sample likely reflects the absence of the natural reservoir host, the bobcat (*Lynx rufus*), from the urban core. However, the risk of introduction remains. The movement of infected domestic cats from endemic regions (e.g., the southern US) could theoretically introduce the pathogen to the local tick population, establishing a novel urban transmission cycle. Our negative findings serve as an important baseline against which future emergence can be measured.

The absence of heartworm antigen should be interpreted with caution. Feline heartworm disease is notoriously difficult to diagnose; cats typically harbor low worm burdens (1–3 worms) and often have single-sex infections that do not produce the antigen detected by commercial assays [29]. Studies utilizing antibody tests often reveal exposure rates significantly higher than antigen prevalence. While our results suggest that heartworm infection is not currently endemic in NYC free-roaming cats, the antigen-only screening approach used here cannot rule out subclinical, single-sex, or pre-patent infection. A further limitation is that we did not perform the modified Knott’s concentration test or an antibody (Ab) ELISA. The Knott’s test was not performed because the small blood volumes obtainable from trapped free-roaming cats had to be prioritized across MAT serology for *T. gondii*, heartworm antigen testing, and qPCR for *Cytauxzoon felis*. In addition, feline heartworm infection is overwhelmingly amicrofilaraemic; naturally infected cats rarely produce detectable circulating microfilariae, owing to low and often single-sex worm burdens and active immune-mediated microfilaricidal activity [29], so antigen ELISA is the recommended primary screen in this host. Nevertheless, best practice combines antigen detection, antibody detection, and microscopic blood examination, and we recommend that future surveillance work in the region incorporate the full diagnostic triad whenever blood volume permits.

The seroprevalence of *T. gondii* (8.9%) in our study was lower than that reported in many other free-roaming cat populations, which can exceed 25% [2,30]. This relatively low rate may reflect an “urban shield” effect, where free-roaming cats in dense cities rely more heavily on anthropogenic food sources (e.g., intentional feeding, restaurant and household waste) than on hunting intermediate hosts like rodents and birds, thereby reducing their trophic exposure to tissue cysts. Seropositivity reflects prior exposure rather than active oocyst shedding; therefore, these findings indicate population-level exposure rather than current environmental contamination risk. Given the high density of cats in the city, even a low shedding rate contributes to a significant cumulative environmental load of oocysts, which can contaminate urban gardens and waterways.

Free-roaming cats are considered sentinels for zoonotic pathogens and environmental contamination in urban settings because they occupy diverse habitats, prey on multiple species, and share public spaces with humans [31,32]. The results from this study suggest that TNR programs should ideally be coupled with parasite control strategies where feasible, although the logistics of treating free-roaming populations remain challenging. Collaboration between veterinarians, public health authorities, municipal park services, and wildlife managers can help map hotspots of contamination, implement targeted interventions, and monitor trends in zoonotic pathogen prevalence and environmental contamination over time.
