# Supplementary material for: Zoonotic endoparasites and Toxoplasma gondii seropositivity in free-roaming cats (Felis catus) from New York City boroughs
Source: PLoS One. 2026 Jul 8;21(7):e0351437. doi: 10.1371/journal.pone.0351437 (PMC13345238; doi:10.1371/journal.pone.0351437)
Supplement: Attachment — Submitted filename: Response to Reviewers.docx [file pone.0351437.s002.docx]

# Response to Reviewers — PONE-D-26-00846

**Manuscript:** Zoonotic endoparasites and *Toxoplasma gondii* seropositivity in free-roaming cats (*Felis catus*) from New York City boroughs.

**Authors:** Nguyen, Gurtowski, Chen, Rosen, Kafle

We thank the Academic Editor and both reviewers for their constructive and supportive evaluations. Their suggestions have definitely helped us to improve the manuscript. We have addressed every point in the revision and provide a point-by-point response below. Reviewer comments are reproduced in *italics*; our responses follow in plain text. Page and line numbers refer to the revised manuscript with tracked changes.

## Response to the Academic Editor / Journal Requirements

1. **PLOS ONE style requirements and file naming.**

The revised manuscript and front matter have been reformatted to follow the PLOS ONE main-body and title-page templates. Files have been renamed per submission guidance: Manuscript.docx, Revised Manuscript with Track Changes.docx, Response to Reviewers.docx, Cover Letter.docx, and Fig1.tif.

**2, 4, 5. Funding statement, Role of Funder, and author–funder relationship.** The funding sentence has been removed from the Acknowledgments. We request that the online funding statement be amended to:

*“This work was supported by institutional startup funds awarded to PK from Long Island University, College of Veterinary Medicine, and from Rowan University, Shreiber School of Veterinary Medicine. The funders provided support in the form of salary for author PK but had no additional role in study design, data collection and analysis, decision to publish, or preparation of the manuscript. The specific roles of all authors are articulated in the ‘author contributions’ section.”*

We confirm in this cover letter that we agree with the standard PLOS funder statement and that the author contributions in the online form reflect each author’s specific role.

**3. Mismatch between Funding Information and Financial Disclosure.** There were no external grant numbers; both awards are unnumbered institutional startup funds. The placeholder grant numbers have been removed from the Funding Information section so that it now matches the Financial Disclosure verbatim.

**6. Data Availability.** We have prepared a complete minimal dataset comprising (i) the de-identified individual-cat dataset used in all analyses, (ii) the R analysis script that reproduces the results. (iv) a data dictionary, and (v) a dual licence (CC-BY 4.0 for data, MIT for code). These files have been deposited at Zenodo (DOI: [https://doi.org/10.5281/zenodo.19866813 ]).

The Data Availability Statement now reads:

*“All de-identified data and analysis code required to reproduce the results are publicly available at Zenodo (https://doi.org/10.5281/zenodo.19866812). Trap-site coordinates have been rounded to two decimal places in the public dataset to protect free-roaming cat colonies; full-precision coordinates are available from the corresponding author on reasonable request, subject to a data-use agreement.”*

**7. Figure 1 copyright (map images).** We agree this is an important compliance issue. The original Figure 1 base layer was generated in Google Earth/Google Maps and is not redistributable under CC-BY 4.0. We have **replaced** Figure 1 entirely with a new map prepared in **QGIS 3.34** using only spatial data and basemap tiles whose licences are compatible with PLOS ONE’s CC-BY 4.0 publication terms:

- New York City borough/county boundaries — New York State GIS Clearinghouse (Counties_Shoreline.shp), public domain.
- U.S. Census Bureau TIGER/Line 2022 state boundary shapefile, public domain.
- Sampling points — our own field-collected coordinates, this study (CC-BY 4.0).
- **OpenStreetMap Standard** XYZ tiles for street/landmark context (© OpenStreetMap contributors, ODbL 1.0).
- **CARTO Positron** XYZ tiles for the muted reference basemap (© CARTO, CC-BY 4.0; underlying data © OpenStreetMap contributors).

Both basemap providers permit use in scholarly publications under their open licences provided the attribution is given in the figure caption, which we have done. The figure caption has been rewritten accordingly. The replacement TIFF meets PLOS ONE’s technical specifications.

**8. Citation of reviewer-recommended works.** Where reviewers recommended specific citations, we evaluated each for relevance and incorporated those that strengthened the manuscript (see point-by-point responses below).

## Response to Reviewer #1

We thank Reviewer #1 for the careful, expert reading and for the specific terminology corrections that have noticeably tightened the manuscript.

**Comment 1.1 — EPG terminology.** *“Unfortunately, the reference to EPG measurement is not appropriate, as no quantitative method (e.g., McMaster) was applied. I understand that an estimation of the relative parasite burden was performed by counting parasite eggs across the entire preparation; however, this is not sufficient to be expressed using the strictly quantitative EPG metric. Therefore, I would recommend removing the EPG measure throughout the manuscript and adopting an ad hoc metric, such as ‘number of eggs per preparation.’”*

We thank the reviewer for raising this methodological point. We respectfully wish to retain the EPG metric, because a clarification of our protocol resolves the concern. The original Methods text was insufficiently detailed and led to an understandable interpretation that only a simple single-step qualitative centrifugal flotation was performed. In fact, fecal samples were processed by the **Wisconsin double-centrifugation flotation technique**, in which a weighed fecal sample (~ 2 g) was mixed with water, strained, centrifuged to pellet the eggs, resuspended in Sheather’s sucrose flotation solution, and centrifuged a second time with a coverslip in place. The Wisconsin double-centrifugation flotation is a concentration method that is widely accepted in veterinary parasitology as a quantitative technique. Because the centrifugation step concentrates and recovers all helminth eggs and protozoal oocysts onto the coverslip, and because we systematically examined the entire 22 × 22 mm coverslip area, every egg recovered by the procedure was enumerated. Dividing this total count by the weight of the fecal sample used (recorded to 0.01 g for every sample) therefore yields a quantitative eggs-per-gram (EPG) value.

To address the reviewer’s concern, we have:

- **Rewritten the Methods (Laboratory analyses) to make the Wisconsin double-centrifugation procedure and weighed-aliquot quantitation explicit, including the formula (EPG = total egg count on coverslip / weight of fecal aliquot in grams) and the relevant references [10, 30, 31].**
- **Clarified that the Wisconsin double-centrifugation flotation is a concentration method that is considered quantitative**, in which the centrifugation step recovers all eggs onto the coverslip and counting the entire coverslip therefore provides a quantitative egg count.
- **Retained “EPG” throughout** as the unit, since the metric is quantitatively justified.
- **Added an explicit Limitations** sentence acknowledging that even quantitative coproscopic egg counts are imperfect proxies for adult-worm burden, given variation in per-female fecundity and intermittent shedding (this also addresses Reviewer #2’s parallel concern, see 2.6).

**Comment 1.2 — Title should reference New York City.** *“I would suggest adding the study area to the title, namely, New York City.”*

Accepted. Since the samples were mostly from the NYC boroughs, the title has been revised to:

*“Zoonotic endoparasites and Toxoplasma gondii seropositivity in free-roaming cats (Felis catus) from the New York City boroughs”*

**Comment 1.3 — Misuse of “Occult Heartworm ELISA test.”** *“Please change ‘Occult Heartworm ELISA test was performed…’ to ‘An ELISA test was performed…’. Note that ‘occult’ refers to a heartworm infection in which microfilariae are not detected in the blood (not investigated in the present study).”*

Accepted. The reviewer is correct; “occult” is a clinical descriptor of amicrofilaraemic infection, not an assay name. The sentence has been revised to:

*“A commercial heartworm antigen ELISA was performed by the Cornell University Animal Health Diagnostic Center as part of their standard diagnostic service to detect circulating Dirofilaria immitis adult-female antigen.”*

We note that the precise commercial kit and reagent details are proprietary to the Cornell AHDC service laboratory and are not publicly disclosed by them; we have therefore cited the AHDC as the assay source rather than naming a kit we cannot independently verify. This wording also follows common practice in veterinary epidemiology papers using AHDC-tested specimens.

**Comment 1.4 — “Variant” → “species.”** *“Line 216. Please change ‘variant’ to ‘species.’”*

Accepted; “canine variant T. canis” has been changed to “canine species *T. canis*.”

**Comment 1.5 — Heartworm wording on line 294.** *“Please change ‘While our results suggest that patent adult heartworm infection is not currently hyper-endemic in NYC feral cats’ to ‘While our results suggest that heartworm infection is not currently endemic in NYC feral cats.’”*

Accepted, with a small qualification we hope the reviewer will agree with: because antigen ELISA detects only patent female adult infection, we have written:

*“While our results suggest that heartworm infection is not currently endemic in NYC free-roaming cats, the antigen-only screening approach used here cannot rule out subclinical, single-sex, or pre-patent infection.”*

This both adopts the reviewer’s recommended phrasing and preserves diagnostic accuracy.

**Comment 1.6 — Knott’s test limitation in the Discussion.** *“Please add to the Discussion the limitation of not performing the Knott’s test… laboratory diagnosis in cats should include all possible tests, i.e. the Knott test, Ag detection and Ab detection.”*

We thank the reviewer for raising this point. We agree that the modified Knott’s test is an important component of the full feline heartworm diagnostic triad, and we have added an explicit limitation paragraph. However, we respectfully note that the omission of the Knott’s test in this study was a deliberate methodological choice driven by sample-volume constraints, and we have explained the rationale in the revised Discussion. Specifically, three considerations drove the decision:

1. **Limited blood volume.** Free-roaming cats trapped under TNR conditions typically yield only small volumes of blood; the available volume had to be allocated across MAT serology for *T. gondii*, antigen ELISA for *D. immitis*, and qPCR for *C. felis*, leaving insufficient volume for the 1 mL of whole blood typically required for the modified Knott’s concentration step.
2. **Cats are usually amicrofilaraemic.** Feline heartworm infection is overwhelmingly amicrofilaraemic; published reviews report that naturally infected cats rarely produce detectable circulating microfilariae owing to low and often single-sex worm burdens and active immune-mediated microfilaricidal activity [29]. Antigen detection is therefore the recommended primary screening test in cats, particularly when sample volume is limiting.
3. **Diagnostic prioritization under field constraints.** Given (1) and (2), we prioritized antigen ELISA as the screening tool most likely to detect a patent adult infection in this small-volume cohort.

We have added the following paragraph to the heartworm subsection of the Discussion:

*“A further limitation is that we did not perform the modified Knott’s concentration test or an antibody (Ab) ELISA. The Knott’s test was not performed because the small blood volumes obtainable from trapped free-roaming cats had to be prioritized across MAT serology for T. gondii, heartworm antigen testing, and qPCR for Cytauxzoon felis. In addition, feline heartworm infection is overwhelmingly amicrofilaraemic; naturally infected cats rarely produce detectable circulating microfilariae, owing to low and often single-sex worm burdens and active immune-mediated microfilaricidal activity [26], so antigen ELISA is the recommended primary screen in this host. Nevertheless, best practice combines antigen detection, antibody detection, and microscopic blood examination, and we recommend that future surveillance work in the region incorporate the full diagnostic triad whenever blood volume permits..”*

We hope this addresses the reviewer’s concern by both acknowledging the limitation and providing a transparent biological rationale for the approach taken.

## Response to Reviewer #2

We thank Reviewer #2 for the positive evaluation and for highlighting the One-Health and public-health framing of the work. The major and minor recommendations have all been incorporated.

**Comment 2.1 — Materials and Methods: DNA extraction kit details.** *“kits used for extracting genetic material from faeces.”*

We thank the reviewer for this important point and agree that complete reagent disclosure is the ideal. We respectfully note, however, that all molecular and antigen-based assays in this study were performed as fee-for-service tests by the Cornell University Animal Health Diagnostic Center (AHDC), a CLIA-certified and AAVLD-accredited veterinary diagnostic laboratory. AHDC uses validated in-house extraction and PCR protocols whose precise reagent components and primer sequences are proprietary to the laboratory and are not publicly disclosed; we therefore cannot provide the exact kit identifiers or primer sequences ourselves. We have, however:

- Revised the Methods (Antigen-based, serologic and molecular testing) to credit AHDC as the assay source, to make explicit that AHDC’s validated in-house protocols were used for DNA extraction and PCR, and to clarify which fecal, serum, and whole-blood subsets were submitted.

The relevant Methods text now reads:

*“Antigen-positive samples were further submitted to the Cornell AHDC for molecular confirmation using their validated in-house PCR assays.*

*Serum samples (n=45) were screened for T. gondii antibodies using the modified agglutination test (MAT), with titers ≥1:25 considered evidence of prior exposure, consistent with previous studies in cats and wildlife [12, 13]. A commercial heartworm antigen ELISA was performed by the Cornell AHDC as part of their standard diagnostic service to detect circulating Dirofilaria immitis adult-female antigen.*

*EDTA whole-blood samples (n=45) were submitted to the Cornell AHDC for detection of Cytauxzoon felis using their validated in-house real-time PCR diagnostic assay.*

We hope this provides sufficient methodological transparency while accurately reflecting that the analyses were conducted in an accredited diagnostic-laboratory setting where complete kit- and primer-level disclosure is not within the authors’ control.

**Comment 2.2 — Describe primers used.** *“Describe the primers adopted in the trials (even in a supplementary file).”*

We thank the reviewer for this request. We respectfully consider that we cannot appropriately provide a primer table in this manuscript, because we did not select or run the primers ourselves. The molecular assays were performed as fee-for-service tests by the Cornell AHDC using their validated in-house assays whose primer sequences and any in-house modifications are proprietary to the laboratory and are not publicly disclosed. We hope the reviewer will agree that this is the most accurate and honest representation of the work performed.

**Comment 2.3 — ELISA test protocol for heartworm.** *“Describe, briefly, the ELISA test protocol for detection of Occult.”*

Addressed jointly with Reviewer #1’s comment 1.3. The misnomer “Occult Heartworm ELISA” has been corrected, and the assay is now described as a commercial heartworm antigen ELISA targeting circulating *D. immitis* adult-female antigen, performed by the Cornell AHDC as part of their standard diagnostic service.

**Comment 2.4 — Add a paragraph on human toxocariasis in the United States and risk to children in public spaces.** *“I suggest the inclusion of a paragraph/sentence concerning human toxocariasis in The United States emphasizing the Human Development Index of New York citizens as well as the high risk of infection by children playing in public places.”*

Accepted. We have added the following paragraph to the Discussion, immediately after the *Toxocara* environmental-contamination paragraph:

*“The public-health implications of these findings are particularly salient in a context such as New York City. Despite NYC’s high Human Development Index and substantial healthcare infrastructure, seroprevalence estimates indicate that approximately 5% of the U.S. population carries antibodies to Toxocara, with markedly higher rates among children, non-Hispanic Black populations, and residents of low-income urban neighborhoods [5]. Toxocariasis is recognized by the U.S. Centers for Disease Control and Prevention as one of five Neglected Parasitic Infections of public-health concern. Children are at disproportionate risk because of geophagia, hand-to-mouth behavior, and play in soil and sand contaminated by free-roaming-cat feces, exposures that are concentrated in dense urban centers where outdoor space is shared between feral colonies and the public. The substantial playground-soil contamination dominated by T. cati that has been documented in NYC public spaces [15], together with the high Toxocara prevalence and the heavy juvenile-male shedding intensity reported here, suggests transmission risk. These findings supports the idea of integrating environmental decontamination, public education, and targeted parasite control into TNR programs as a One-Health priority.*

**Comment 2.5 — Conclusion.** *“I would be glad to verify the conclusion after the revised version of the manuscript, considering the recommendations.”*

The Conclusion has been revised to reflect: (i) the clarified Wisconsin double-centrifugation methodology and the validity of the EPG metric, (ii) the diagnostic-triad recommendation for future feline heartworm work, (iii) the pediatric-public-health framing of Toxocara environmental contamination, and (iv) the call for continued surveillance for emerging vector-borne pathogens such as *Cytauxzoon felis* given the documented expansion of *Amblyomma americanum* in the region.

**Comment 2.6 — Egg count vs. frequency of infected cats.** *“In our opinion, the epg count is less relevant than the frequency of infected cats. A unique T. cati for example, may shed a high number of eggs/day (more than 100,000). Then, presence of eggs may not mirror the infection burden of parasites.”*

We thank the reviewer for this thoughtful comment. We agree completely that egg counts are not direct estimates of adult-worm burden: per-female *T. cati* fecundity can exceed 100,000 eggs/day, fecundity varies with worm age and immune status, and shedding is intermittent and density-dependent. We have added an explicit caveat to the Discussion, supported by an appropriate citation, to make this clear. We wish to retain, but contextualize, the intensity analysis, for the following reason:

The central public-health question this study addresses is **environmental contamination load**, not within-host worm burden. From an environmental-transmission and One-Health standpoint, eggs deposited into the urban environment are precisely what matters for human, pet, and wildlife exposure — regardless of whether they originate from one fecund female or several. The ten-fold difference in egg-shedding intensity between juveniles and adults, and the nearly four-fold sex difference, identify a small subset of cats that disproportionately drive environmental egg deposition. This information is operationally useful for designing **targeted** deworming strategies within TNR programs (i.e., prioritizing juveniles and males during routine TNR procedures), which would not be possible from prevalence data alone.

The new caveat sentence in the revised Discussion reads:

*“Egg-output measures should be interpreted as indices of environmental contamination potential rather than direct estimates of adult-worm burden, since per-female fecundity in Toxocara cati can exceed 100,000 eggs per day and is modulated by immune status, worm age, and density-dependent effects [32].”*

We therefore respectfully retain the EPG analysis but have rewritten the relevant paragraph to align with the reviewer’s caution against over-interpretation. We hope this represents a satisfactory middle ground.

**Comment 2.7 — General positive remarks on the dataset.** *“…the number of infected cats is a surprisingly and undoubtedly an ‘extraordinary’ result in a developed country.”*

We thank the reviewer for this assessment. We have strengthened the Discussion to emphasize the comparative point that our 54% *Toxocara* prevalence aligns more closely with reports from low- and middle-income settings than with most surveys from high-HDI cities, and we link this directly to the pediatric-public-health framing requested in Comment 2.4.

## Summary of Changes

The complete list of changes is provided in the Revised Manuscript with Track Changes. Key revisions include: (i) revised title; (ii) expanded Materials and Methods section that explicitly describes the Wisconsin double-centrifugation flotation protocol and weighed-aliquot EPG calculation; (iii) revised heartworm-assay description and a new Discussion paragraph explaining the rationale for omitting the Knott’s test together with the recommendation that future work apply the full diagnostic triad; (iv) new Discussion paragraph on pediatric toxocariasis, Toxocara playground contamination, and the One-Health framing in NYC; (v) added caveat that EPG is an index of environmental contamination potential rather than an estimate of adult-worm burden; (vi) replacement of Figure 1 with a copyright-compliant map prepared in QGIS 3.34 over public-domain shapefiles; (vii) revised Acknowledgments, Funding, and Data Availability statements; (viii) deposition of the de-identified minimal dataset and analysis code on Zenodo,; and (ix) revised molecular-methods description to credit the Cornell AHDC as the accredited assay source.

We hope the editor and reviewers find these revisions responsive and that the manuscript is now suitable for publication in *PLOS ONE*.

Sincerely,

**Pratap Kafle,** on behalf of all authors

Shreiber School of Veterinary Medicine, Rowan University

kafle@rowan.edu

## Cited references new to this revision

The following references have been added to the manuscript bibliography (numbered to follow the existing reference list):

- **[30] Dryden MW, Payne PA, Ridley R, Smith V. Comparison of common fecal flotation techniques for the recovery of parasite eggs and oocysts. Vet Ther. 2005;6(1):15–28.**
- **[31] Cringoli G, Maurelli MP, Levecke B, Bosco A, Vercruysse J, Utzinger J, et al. The Mini-FLOTAC technique for the diagnosis of helminth and protozoan infections in humans and animals. Nat Protoc. 2017;12(9):1723–1732. doi: 10.1038/nprot.2017.067.**
- **[32] Overgaauw PAM, van Knapen F. Veterinary and public health aspects of Toxocara spp. Vet Parasitol. 2013;193(4):398–403. doi: 10.1016/j.vetpar.2012.12.035.**
