# Supplementary material for: Zoonotic endoparasites and Toxoplasma gondii seropositivity in free-roaming cats (Felis catus) from New York City boroughs
Source: PLoS One. 2026 Jul 8;21(7):e0351437. doi: 10.1371/journal.pone.0351437 (PMC13345238; doi:10.1371/journal.pone.0351437)
Supplement: Peer review history

# Peer review: Decision Letter 0

**DOI:** 10.1371/journal.pone.0351437.r001

**Authors:** Balbir B. Singh

24 Apr 2026

PONE-D-26-00846

Zoonotic endoparasites and *Toxoplasma gondii*   seropositivity in free-roaming cats (*Felis catus*  ) from an urban environment

PLOS One

Dear Dr. Kafle,

Thank you for submitting your manuscript to PLOS ONE. After careful consideration, we feel that it has merit but does not fully meet PLOS ONE’s publication criteria as it currently stands. Therefore, we invite you to submit a revised version of the manuscript that addresses the points raised during the review process.

==============================

Please revise the manuscript as per reviewer comments.

==============================

Please submit your revised manuscript by Jun 08 2026 11:59PM. If you will need more time than this to complete your revisions, please reply to this message or contact the journal office at plosone@plos.org. When you're ready to submit your revision, log on to https://www.editorialmanager.com/pone/ and select the 'Submissions Needing Revision' folder to locate your manuscript file.

Please include the following items when submitting your revised manuscript:

- A letter that responds to each point raised by the academic editor and reviewer(s). You should upload this letter as a separate file labeled 'Response to Reviewers'.
- A marked-up copy of your manuscript that highlights changes made to the original version. You should upload this as a separate file labeled 'Revised Manuscript with Track Changes'.
- An unmarked version of your revised paper without tracked changes. You should upload this as a separate file labeled 'Manuscript'.

If you would like to make changes to your financial disclosure, please include your updated statement in your cover letter. Guidelines for resubmitting your figure files are available below the reviewer comments at the end of this letter.

If applicable, we recommend that you deposit your laboratory protocols in protocols.io to enhance the reproducibility of your results. Protocols.io assigns your protocol its own identifier (DOI) so that it can be cited independently in the future. For instructions see: https://journals.plos.org/plosone/s/submission-guidelines#loc-laboratory-protocols. Additionally, PLOS ONE offers an option for publishing peer-reviewed Lab Protocol articles, which describe protocols hosted on protocols.io. Read more information on sharing protocols at https://plos.org/protocols?utm_medium=editorial-email&utm_source=authorletters&utm_campaign=protocols.

As the corresponding author, your ORCID iD is verified in the submission system and will appear in the published article. PLOS supports the use of ORCID, and we encourage all coauthors to register for an ORCID iD and use it as well. Please encourage your coauthors to verify their ORCID iD within the submission system before final acceptance, as unverified ORCID iDs will not appear in the published article. *Only*   the individual author can complete the verification step; PLOS staff *cannot*   verify ORCID iDs on behalf of authors.

We look forward to receiving your revised manuscript.

Kind regards,

Balbir B. Singh, Ph. D

Academic Editor

PLOS One

Journal Requirements:

When submitting your revision, we need you to address these additional requirements.

1. Please ensure that your manuscript meets PLOS ONE's style requirements, including those for file naming. The PLOS ONE style templates can be found at

https://journals.plos.org/plosone/s/file?id=wjVg/PLOSOne_formatting_sample_main_body.pdf and

https://journals.plos.org/plosone/s/file?id=ba62/PLOSOne_formatting_sample_title_authors_affiliations.pdf

2. Thank you for stating the following in the Acknowledgments Section of your manuscript:

“This study was supported by startup funds from Long Island University College of Veterinary Medicine, and Shreiber School of Veterinary Medicine.”

We note that you have provided funding information that is not currently declared in your Funding Statement. However, funding information should not appear in the Acknowledgments section or other areas of your manuscript. We will only publish funding information present in the Funding Statement section of the online submission form.

Please remove any funding-related text from the manuscript and let us know how you would like to update your Funding Statement. Currently, your Funding Statement reads as follows:

“This work was funded by institutional startup funds by Long Island University, College of Veterinary Medicine and Rowan university, Shreiber School of Veterinary Medicine”

Please include your amended statements within your cover letter; we will change the online submission form on your behalf.

3. We note that the grant information you provided in the ‘Funding Information’ and ‘Financial Disclosure’ sections do not match.

When you resubmit, please ensure that you provide the correct grant numbers for the awards you received for your study in the ‘Funding Information’ section.

4. Thank you for stating the following financial disclosure:

“This work was funded by institutional startup funds by Long Island University, College of Veterinary Medicine and Rowan university, Shreiber School of Veterinary Medicine”

Please state what role the funders took in the study.  If the funders had no role, please state: "The funders had no role in study design, data collection and analysis, decision to publish, or preparation of the manuscript."

If this statement is not correct you must amend it as needed.

Please include this amended Role of Funder statement in your cover letter; we will change the online submission form on your behalf.

5. Thank you for stating the following financial disclosure:

“This work was funded by institutional startup funds by Long Island University, College of Veterinary Medicine and Rowan university, Shreiber School of Veterinary Medicine”

We note that one or more of the authors is affiliated with the funding organization, indicating the funder may have had some role in the design, data collection, analysis or preparation of your manuscript for publication; in other words, the funder played an indirect role through the participation of the co-authors. If the funding organization did not play a role in the study design, data collection and analysis, decision to publish, or preparation of the manuscript and only provided financial support in the form of authors' salaries and/or research materials, please do the following:

1. Review your statements relating to the author contributions, and ensure you have specifically and accurately indicated the role(s) that these authors had in your study. These amendments should be made in the online form.

2. Confirm in your cover letter that you agree with the following statement, and we will change the online submission form on your behalf:

“The funder provided support in the form of salaries for authors [insert relevant initials], but did not have any additional role in the study design, data collection and analysis, decision to publish, or preparation of the manuscript. The specific roles of these authors are articulated in the ‘author contributions’ section.

6. We note that your Data Availability Statement is currently as follows: “All relevant data are within the manuscript and its Supporting Information files.”

Please confirm at this time whether or not your submission contains all raw data required to replicate the results of your study. Authors must share the “minimal data set” for their submission. PLOS defines the minimal data set to consist of the data required to replicate all study findings reported in the article, as well as related metadata and methods (https://journals.plos.org/plosone/s/data-availability#loc-minimal-data-set-definition).

For example, authors should submit the following data:

- The values behind the means, standard deviations and other measures reported;

- The values used to build graphs;

- The points extracted from images for analysis.

Authors do not need to submit their entire data set if only a portion of the data was used in the reported study.

If your submission does not contain these data, please either upload them as Supporting Information files or deposit them to a stable, public repository and provide us with the relevant URLs, DOIs, or accession numbers. For a list of recommended repositories, please see https://journals.plos.org/plosone/s/recommended-repositories.

If there are ethical or legal restrictions on sharing a de-identified data set, please explain them in detail (e.g., data contain potentially sensitive information, data are owned by a third-party organization, etc.) and who has imposed them (e.g., an ethics committee). Please also provide contact information for a data access committee, ethics committee, or other institutional body to which data requests may be sent. If data are owned by a third party, please indicate how others may request data access.

7. We note that Figure 1 in your submission contain map images which may be copyrighted. All PLOS content is published under the Creative Commons Attribution License (CC BY 4.0), which means that the manuscript, images, and Supporting Information files will be freely available online, and any third party is permitted to access, download, copy, distribute, and use these materials in any way, even commercially, with proper attribution. For these reasons, we cannot publish previously copyrighted maps or satellite images created using proprietary data, such as Google software (Google Maps, Street View, and Earth). For more information, see our copyright guidelines: http://journals.plos.org/plosone/s/licenses-and-copyright.

We require you to either (1) present written permission from the copyright holder to publish these figures specifically under the CC BY 4.0 license, or (2) remove the figures from your submission:

1. You may seek permission from the original copyright holder of Figure(s) [#] to publish the content specifically under the CC BY 4.0 license.

We recommend that you contact the original copyright holder with the Content Permission Form (http://journals.plos.org/plosone/s/file?id=7c09/content-permission-form.pdf) and the following text:

“I request permission for the open-access journal PLOS ONE to publish XXX under the Creative Commons Attribution License (CCAL) CC BY 4.0 (http://creativecommons.org/licenses/by/4.0/). Please be aware that this license allows unrestricted use and distribution, even commercially, by third parties. Please reply and provide explicit written permission to publish XXX under a CC BY license and complete the attached form.”

Please upload the completed Content Permission Form or other proof of granted permissions as an "Other" file with your submission.

In the figure caption of the copyrighted figure, please include the following text: “Reprinted from [ref] under a CC BY license, with permission from [name of publisher], original copyright [original copyright year].”

2. If you are unable to obtain permission from the original copyright holder to publish these figures under the CC BY 4.0 license or if the copyright holder’s requirements are incompatible with the CC BY 4.0 license, please either i) remove the figure or ii) supply a replacement figure that complies with the CC BY 4.0 license. Please check copyright information on all replacement figures and update the figure caption with source information. If applicable, please specify in the figure caption text when a figure is similar but not identical to the original image and is therefore for illustrative purposes only.

The following resources for replacing copyrighted map figures may be helpful:

USGS National Map Viewer (public domain): http://viewer.nationalmap.gov/viewer/

The Gateway to Astronaut Photography of Earth (public domain): http://eol.jsc.nasa.gov/sseop/clickmap/

Maps at the CIA (public domain): https://www.cia.gov/library/publications/the-world-factbook/index.html and https://www.cia.gov/library/publications/cia-maps-publications/index.html

NASA Earth Observatory (public domain): http://earthobservatory.nasa.gov/

Landsat: http://landsat.visibleearth.nasa.gov/

USGS EROS (Earth Resources Observatory and Science (EROS) Center) (public domain): http://eros.usgs.gov/#

Natural Earth (public domain): http://www.naturalearthdata.com/

8. If the reviewer comments include a recommendation to cite specific previously published works, please review and evaluate these publications to determine whether they are relevant and should be cited. There is no requirement to cite these works unless the editor has indicated otherwise.

[Note: HTML markup is below. Please do not edit.]

Reviewers' comments:

Reviewer's Responses to Questions

**Comments to the Author**

1. Is the manuscript technically sound, and do the data support the conclusions?

The manuscript must describe a technically sound piece of scientific research with data that supports the conclusions. Experiments must have been conducted rigorously, with appropriate controls, replication, and sample sizes. The conclusions must be drawn appropriately based on the data presented.

Reviewer #1: Partly

Reviewer #2: Yes

**********

2. Has the statistical analysis been performed appropriately and rigorously?

Reviewer #1: Yes

Reviewer #2: Yes

**********

3. Have the authors made all data underlying the findings in their manuscript fully available?

The PLOS Data policy requires authors to make all data underlying the findings described in their manuscript fully available without restriction, with rare exception (please refer to the Data Availability Statement in the manuscript PDF file). The data should be provided as part of the manuscript or its supporting information, or deposited to a public repository. For example, in addition to summary statistics, the data points behind means, medians and variance measures should be available. If there are restrictions on publicly sharing data—e.g. participant privacy or use of data from a third party—those must be specified.

Reviewer #1: Yes

Reviewer #2: Yes

**********

4. Is the manuscript presented in an intelligible fashion and written in standard English?

PLOS ONE does not copyedit accepted manuscripts, so the language in submitted articles must be clear, correct, and unambiguous. Any typographical or grammatical errors should be corrected at revision, so please note any specific errors here.

Reviewer #1: Yes

Reviewer #2: Yes

**********

5. Review Comments to the Author

Please use the space provided to explain your answers to the questions above. You may also include additional comments for the author, including concerns about dual publication, research ethics, or publication ethics. (Please upload your review as an attachment if it exceeds 20,000 characters)

Reviewer #1: Dear Authors,

Your manuscript entitled “Zoonotic endoparasites and Toxoplasma gondii seropositivity in free-roaming cats (Felis catus) from an urban environment” is a valuable contribution to the necessary surveillance of parasitic infections in animals living in urban settings. Studies of this type should be conducted regularly in order to accumulate important epidemiological data.

I believe that your manuscript is worthy of publication after addressing the following points:

1. Unfortunately, the reference to EPG measurement is not appropriate, as no quantitative method (e.g., McMaster) was applied. I understand that an estimation of the relative parasite burden was performed by counting parasite eggs across the entire preparation; however, this is not sufficient to be expressed using the strictly quantitative EPG metric. Therefore, I would recommend removing the EPG measure throughout the manuscript and, to utilise your approximate estimate of parasite burden, adopting an ad hoc metric, such as “number of eggs per preparation.”

2. I would suggest adding the study area to the title, namely, New York City. This is simply a suggestion, and I leave it to your judgment and preference.

3. Line 118. Please change “Occult Heartworm ELISA test was performed…” to “An ELISA test was performed…”. Note that “occult” refers to a heartworm infection in which microfilariae are not detected in the blood (not investigated in the present study).

4. Line 216. Please change “variant” to “species”.

5. Line 294. Please change “While our results suggest that patent adult heartworm infection is not currently hyper-endemic in NYC feral cats” to “While our results suggest that heartworm infection is not currently endemic in NYC feral cats.”

6. Please add to the Discussion the limitation of not performing the Knott’s test in the investigation of heartworm infection. Although heartworm infection in cats is typically amicrofilaraemic (i.e., occult), microfilaraemia does occur. Given that, a) the number of heartworms in cats is usually very low, b) that a single mature pair of adult heartworms can produce microfilariae, but c) would most probably result in a negative serological test (too little antigen to be detected), laboratory diagnosis in cats should include all possible tests, i.e. the Knott test, Ag detection and Ab detection.

Reviewer #2: The Manuscript Number PONE-D-26-00846, entitled “Zoonotic endoparasites and Toxoplasma gondii seropositivity in free-roaming cats (Felis catus) from an urban environment” show interesting and relevant results on zoonotic endoparasites in New York, particularly a high number of infected animals by Toxocara cati. The results, considering the results, reinforce the strategies employed to deal with parasites of free-roaming cats. However, authors are recommended to carefully revise and to include some information.

I hereby would like to present some recommendations for improvement of the current version of the manuscript.

Major comments

## Materials and Methods

Please, give some details regard to the following procedures:

- kits used for extracting genetic material from faeces.

- Describe the primers adopted in the trials (even in a supplementary file).

- Describe, briefly, the ELISA test protocol for detection of Occult.

## Discussion

Despite the main aim of the researchers was to diagnostic zoonotic endoparasites in free-roaming cats, I suggest the inclusion of a paragraph/sentence concerning human toxocariasis in The United States emphasizing the Human Development Index of |New York citizens as well as the high risk of infection by children playing in public places.

## Conclusion

I would be glad to verify the conclusion after the revised version of the manuscript, considering the recommendations.

Minor comments

In our opinion, the epg count is less relevant than the frequency of infected cats. A unique T. cati for example, may shed a high number of eggs/day (more than 100.000). Then, presence of eggs may not mirror the infection burden of parasites. Nevertheless, the number of infected cats is a surprisingly and undoubtedly an “extraordinary” result in a developed country.

**********

6. PLOS authors have the option to publish the peer review history of their article (what does this mean?). If published, this will include your full peer review and any attached files.

If you choose “no”, your identity will remain anonymous but your review may still be made public.

**Do you want your identity to be public for this peer review?**   For information about this choice, including consent withdrawal, please see our Privacy Policy.

Reviewer #1: No

Reviewer #2: No

**********

[NOTE: If reviewer comments were submitted as an attachment file, they will be attached to this email and accessible via the submission site. Please log into your account, locate the manuscript record, and check for the action link "View Attachments". If this link does not appear, there are no attachment files.]

To ensure your figures meet our technical requirements, please review our figure guidelines: https://journals.plos.org/plosone/s/figures

You may also use PLOS’s free figure tool, NAAS, to help you prepare publication quality figures: https://journals.plos.org/plosone/s/figures#loc-tools-for-figure-preparation.

NAAS will assess whether your figures meet our technical requirements by comparing each figure against our figure specifications.

# Author comment: Author response to Decision Letter 1

**DOI:** 10.1371/journal.pone.0351437.r002

12 May 2026

Response to the Academic Editor / Journal Requirements

1. PLOS ONE style requirements and file naming.

The revised manuscript and front matter have been reformatted to follow the PLOS ONE main-body and title-page templates. Files have been renamed per submission guidance: Manuscript.docx, Revised Manuscript with Track Changes.docx, Response to Reviewers.docx, Cover Letter.docx, and Fig1.tif.

2, 4, 5. Funding statement, Role of Funder, and author–funder relationship. The funding sentence has been removed from the Acknowledgments. We request that the online funding statement be amended to:

“This work was supported by institutional startup funds awarded to PK from Long Island University, College of Veterinary Medicine, and from Rowan University, Shreiber School of Veterinary Medicine. The funders provided support in the form of salary for author PK but had no additional role in study design, data collection and analysis, decision to publish, or preparation of the manuscript. The specific roles of all authors are articulated in the ‘author contributions’ section.”

We confirm in this cover letter that we agree with the standard PLOS funder statement and that the author contributions in the online form reflect each author’s specific role.

3. Mismatch between Funding Information and Financial Disclosure. There were no external grant numbers; both awards are unnumbered institutional startup funds. The placeholder grant numbers have been removed from the Funding Information section so that it now matches the Financial Disclosure verbatim.

6. Data Availability. We have prepared a complete minimal dataset comprising (i) the de-identified individual-cat dataset used in all analyses, (ii) the R analysis script that reproduces the results. (iv) a data dictionary, and (v) a dual licence (CC-BY 4.0 for data, MIT for code). These files have been deposited at Zenodo (DOI: [https://doi.org/10.5281/zenodo.19866813 ]).

The Data Availability Statement now reads:

“All de-identified data and analysis code required to reproduce the results are publicly available at Zenodo (https://doi.org/10.5281/zenodo.19866812). Trap-site coordinates have been rounded to two decimal places in the public dataset to protect free-roaming cat colonies; full-precision coordinates are available from the corresponding author on reasonable request, subject to a data-use agreement.”

7. Figure 1 copyright (map images). We agree this is an important compliance issue. The original Figure 1 base layer was generated in Google Earth/Google Maps and is not redistributable under CC-BY 4.0. We have replaced Figure 1 entirely with a new map prepared in QGIS 3.34 using only spatial data and basemap tiles whose licences are compatible with PLOS ONE’s CC-BY 4.0 publication terms:

• New York City borough/county boundaries — New York State GIS Clearinghouse (Counties_Shoreline.shp), public domain.

• U.S. Census Bureau TIGER/Line 2022 state boundary shapefile, public domain.

• Sampling points — our own field-collected coordinates, this study (CC-BY 4.0).

• OpenStreetMap Standard XYZ tiles for street/landmark context (© OpenStreetMap contributors, ODbL 1.0).

• CARTO Positron XYZ tiles for the muted reference basemap (© CARTO, CC-BY 4.0; underlying data © OpenStreetMap contributors).

Both basemap providers permit use in scholarly publications under their open licences provided the attribution is given in the figure caption, which we have done. The figure caption has been rewritten accordingly. The replacement TIFF meets PLOS ONE’s technical specifications.

8. Citation of reviewer-recommended works. Where reviewers recommended specific citations, we evaluated each for relevance and incorporated those that strengthened the manuscript (see point-by-point responses below).

Response to Reviewer #1

We thank Reviewer #1 for the careful, expert reading and for the specific terminology corrections that have noticeably tightened the manuscript.

Comment 1.1 — EPG terminology. “Unfortunately, the reference to EPG measurement is not appropriate, as no quantitative method (e.g., McMaster) was applied. I understand that an estimation of the relative parasite burden was performed by counting parasite eggs across the entire preparation; however, this is not sufficient to be expressed using the strictly quantitative EPG metric. Therefore, I would recommend removing the EPG measure throughout the manuscript and adopting an ad hoc metric, such as ‘number of eggs per preparation.’”

We thank the reviewer for raising this methodological point. We respectfully wish to retain the EPG metric, because a clarification of our protocol resolves the concern. The original Methods text was insufficiently detailed and led to an understandable interpretation that only a simple single-step qualitative centrifugal flotation was performed. In fact, fecal samples were processed by the Wisconsin double-centrifugation flotation technique, in which a weighed fecal sample (~ 2 g) was mixed with water, strained, centrifuged to pellet the eggs, resuspended in Sheather’s sucrose flotation solution, and centrifuged a second time with a coverslip in place. The Wisconsin double-centrifugation flotation is a concentration method that is widely accepted in veterinary parasitology as a quantitative technique. Because the centrifugation step concentrates and recovers all helminth eggs and protozoal oocysts onto the coverslip, and because we systematically examined the entire 22 × 22 mm coverslip area, every egg recovered by the procedure was enumerated. Dividing this total count by the weight of the fecal sample used (recorded to 0.01 g for every sample) therefore yields a quantitative eggs-per-gram (EPG) value.

To address the reviewer’s concern, we have:

• Rewritten the Methods (Laboratory analyses) to make the Wisconsin double-centrifugation procedure and weighed-aliquot quantitation explicit, including the formula (EPG = total egg count on coverslip / weight of fecal aliquot in grams) and the relevant references [10, 30, 31].

• Clarified that the Wisconsin double-centrifugation flotation is a concentration method that is considered quantitative, in which the centrifugation step recovers all eggs onto the coverslip and counting the entire coverslip therefore provides a quantitative egg count.

• Retained “EPG” throughout as the unit, since the metric is quantitatively justified.

• Added an explicit Limitations sentence acknowledging that even quantitative coproscopic egg counts are imperfect proxies for adult-worm burden, given variation in per-female fecundity and intermittent shedding (this also addresses Reviewer #2’s parallel concern, see 2.6).

Comment 1.2 — Title should reference New York City. “I would suggest adding the study area to the title, namely, New York City.”

Accepted. Since the samples were mostly from the NYC boroughs, the title has been revised to:

“Zoonotic endoparasites and Toxoplasma gondii seropositivity in free-roaming cats (Felis catus) from the New York City boroughs”

Comment 1.3 — Misuse of “Occult Heartworm ELISA test.” “Please change ‘Occult Heartworm ELISA test was performed…’ to ‘An ELISA test was performed…’. Note that ‘occult’ refers to a heartworm infection in which microfilariae are not detected in the blood (not investigated in the present study).”

Accepted. The reviewer is correct; “occult” is a clinical descriptor of amicrofilaraemic infection, not an assay name. The sentence has been revised to:

“A commercial heartworm antigen ELISA was performed by the Cornell University Animal Health Diagnostic Center as part of their standard diagnostic service to detect circulating Dirofilaria immitis adult-female antigen.”

We note that the precise commercial kit and reagent details are proprietary to the Cornell AHDC service laboratory and are not publicly disclosed by them; we have therefore cited the AHDC as the assay source rather than naming a kit we cannot independently verify. This wording also follows common practice in veterinary epidemiology papers using AHDC-tested specimens.

Comment 1.4 — “Variant” → “species.” “Line 216. Please change ‘variant’ to ‘species.’”

Accepted; “canine variant T. canis” has been changed to “canine species T. canis.”

Comment 1.5 — Heartworm wording on line 294. “Please change ‘While our results suggest that patent adult heartworm infection is not currently hyper-endemic in NYC feral cats’ to ‘While our results suggest that heartworm infection is not currently endemic in NYC feral cats.’”

Accepted, with a small qualification we hope the reviewer will agree with: because antigen ELISA detects only patent female adult infection, we have written:

“While our results suggest that heartworm infection is not currently endemic in NYC free-roaming cats, the antigen-only screening approach used here cannot rule out subclinical, single-sex, or pre-patent infection.”

This both adopts the reviewer’s recommended phrasing and preserves diagnostic accuracy.

Comment 1.6 — Knott’s test limitation in the Discussion. “Please add to the Discussion the limitation of not performing the Knott’s test… laboratory diagnosis in cats should include all possible tests, i.e. the Knott test, Ag detection and Ab detection.”

We thank the reviewer for raising this point. We agree that the modified Knott’s test is an important component of the full feline heartworm diagnostic triad, and we have added an explicit limitation paragraph. However, we respectfully note that the omission of the Knott’s test in this study was a deliberate methodological choice driven by sample-volume constraints, and we have explained the rationale in the revised Discussion. Specifically, three considerations drove the decision:

1. Limited blood volume. Free-roaming cats trapped under TNR conditions typically yield only small volumes of blood; the available volume had to be allocated across MAT serology for T. gondii, antigen ELISA for D. immitis, and qPCR for C. felis, leaving insufficient volume for the 1 mL of whole blood typically required for the modified Knott’s concentration step.

2. Cats are usually amicrofilaraemic. Feline heartworm infection is overwhelmingly amicrofilaraemic; published reviews report that naturally infected cats rarely produce detectable circulating microfilariae owing to low and often single-sex worm burdens and active immune-mediated microfilaricidal activity [29]. Antigen detection is therefore the recommended primary screening test in cats, particularly when sample volume is limiting.

3. Diagnostic prioritization under field constraints. Given (1) and (2), we prioritized antigen ELISA as the screening tool most likely to detect a patent adult infection in this small-volume cohort.

We have added the following paragraph to the heartworm subsection of the Discussion:

“A further limitation is that we did not perform the modified Knott’s concentration test or an antibody (Ab) ELISA. The Knott’s test was not performed because the small blood volumes obtainable from trapped free-roaming cats had to be prioritized across MAT serology for T. gondii, heartworm antigen testing, and qPCR for Cytauxzoon felis. In addition, feline heartworm infection is overwhelmingly amicrofilaraemic; naturally infected cats rarely produce detectable circulating microfilariae, owing to low and often single-sex worm burdens and active immune-mediated microfilaricidal activity [26], so antigen ELISA is the recommended primary screen in this host. Nevertheless, best practice combines antigen detection, antibody detection, and microscopic blood examination, and we recommend that future surveillance work in the region incorporate the full diagnostic triad whenever blood volume permits..”

We hope this addresses the reviewer’s concern by both acknowledging the limitation and providing a transparent biological rationale for the approach taken.

Response to Reviewer #2

We thank Reviewer #2 for the positive evaluation and for highlighting the One-Health and public-health framing of the work. The major and minor recommendations have all been incorporated.

Comment 2.1 — Materials and Methods: DNA extraction kit details. “kits used for extracting genetic material from faeces.”

We thank the reviewer for this important point and agree that complete reagent disclosure is the ideal. We respectfully note, however, that all molecular and antigen-based assays in this study were performed as fee-for-service tests by the Cornell University Animal Health Diagnostic Center (AHDC), a CLIA-certified and AAVLD-accredited veterinary diagnostic laboratory. AHDC uses validated in-house extraction and PCR protocols whose precise reagent components and primer sequences are proprietary to the laboratory and are not publicly disclosed; we therefore cannot provide the exact kit identifiers or primer sequences ourselves. We have, however:

• Revised the Methods (Antigen-based, serologic and molecular testing) to credit AHDC as the assay source, to make explicit that AHDC’s validated in-house protocols were used for DNA extraction and PCR, and to clarify which fecal, serum, and whole-blood subsets were submitted.

The relevant Methods text now reads:

“Antigen-positive samples were further submitted to the Cornell AHDC for molecular confirmation using their validated in-house PCR assays.

Serum samples (n=45) were screened for T. gondii antibodies using the modified agglutination test (MAT), with titers ≥1:25 considered evidence of prior exposure, consistent with previous studies in cats and wildlife [12, 13]. A commercial heartworm antigen ELISA was performed by the Cornell AHDC as part of their standard diagnostic service to detect circulating Dirofilaria immitis adult-female antigen.

EDTA whole-blood samples (n=45) were submitted to the Cornell AHDC for detection of Cytauxzoon felis using their validated in-house real-time PCR diagnostic assay.

We hope this provides sufficient methodological transparency while accurately reflecting that the analyses were conducted in an accredited diagnostic-laboratory setting where complete kit- and primer-level disclosure is not within the authors’ control.

Comment 2.2 — Describe primers used. “Describe the primers adopted in the trials (even in a supplementary file).”

We thank the reviewer for this request. We respectfully consider that we cannot appropriately provide a primer table in this manuscript, because we did not select or run the primers ourselves. The molecular assays were performed as fee-for-service tests by the Cornell AHDC using their validated in-house assays whose primer sequences and any in-house modifications are proprietary to the laboratory and are not publicly disclosed. We hope the reviewer will agree that this is the most accurate and honest representation of the work performed.

Comment 2.3 — ELISA test protocol for heartworm. “Describe, briefly, the ELISA test protocol for detection of Occult.”

Addressed jointly with Reviewer #1’s comment 1.3. The misnomer “Occult Heartworm ELISA” has been corrected, and the assay is now described as a commercial heartworm antigen ELISA targeting circulating D. immitis adult-female antigen, performed by the Cornell AHDC as part of their standard diagnostic service.

Comment 2.4 — Add a paragraph on human toxocariasis in the United States and risk to children in public spaces. “I suggest the inclusion of a paragraph/sentence concerning human toxocariasis in The United States emphasizing the Human Development Index of New York citizens as well as the high risk of infection by children playing in public places.”

Accepted. We have added the following paragraph to the Discussion, immediately after the Toxocara environmental-contamination paragraph:

“The public-health implications of these findings are particularly salient in a context such as New York City. Despite NYC’s high Human Development Index and substantial healthcare infrastructure, seroprevalence estimates indicate that approximately 5% of the U.S. population carries antibodies to Toxocara, with markedly higher rates among children, non-Hispanic Black populations, and residents of low-income urban neighborhoods [5]. Toxocariasis is recognized by the U.S. Centers for Disease Control and Prevention as one of five Neglected Parasitic Infections of public-health concern. Children are at disproport

Attachment Submitted filename: Response to Reviewers.docx

# Editor report: Decision Letter 1

**DOI:** 10.1371/journal.pone.0351437.r003

**Authors:** Balbir B. Singh

28 May 2026

Zoonotic endoparasites and *Toxoplasma gondii*   seropositivity in free-roaming cats (*Felis catus*  ) from New York City Boroughs

PONE-D-26-00846R1

Dear Dr. Kafle,

We’re pleased to inform you that your manuscript has been judged scientifically suitable for publication and will be formally accepted for publication once it meets all outstanding technical requirements.

Within one week, you’ll receive an e-mail detailing the required amendments. When these have been addressed, you’ll receive a formal acceptance letter and your manuscript will be scheduled for publication.

An invoice will be generated when your article is formally accepted. Please note, if your institution has a publishing partnership with PLOS and your article meets the relevant criteria, all or part of your publication costs will be covered. Please make sure your user information is up-to-date by logging into Editorial Manager at Editorial Manager® and clicking the ‘Update My Information' link at the top of the page. For questions related to billing, please contact billing support.

If your institution or institutions have a press office, please notify them about your upcoming paper to help maximize its impact. If they’ll be preparing press materials, please inform our press team as soon as possible -- no later than 48 hours after receiving the formal acceptance. Your manuscript will remain under strict press embargo until 2 pm Eastern Time on the date of publication. For more information, please contact onepress@plos.org.

Kind regards,

Balbir B. Singh, Ph. D

Academic Editor

PLOS One

Additional Editor Comments (optional):

Reviewers' comments:

# Editor report: Acceptance letter

**DOI:** 10.1371/journal.pone.0351437.r004

**Authors:** Balbir B. Singh

PONE-D-26-00846R1

PLOS One

Dear Dr. Kafle,

I'm pleased to inform you that your manuscript has been deemed suitable for publication in PLOS One. Congratulations! Your manuscript is now being handed over to our production team.

At this stage, our production department will prepare your paper for publication. This includes ensuring the following:

* All references, tables, and figures are properly cited

* All relevant supporting information is included in the manuscript submission,

* There are no issues that prevent the paper from being properly typeset

You will receive further instructions from the production team, including instructions on how to review your proof when it is ready. Please keep in mind that we are working through a large volume of accepted articles, so please give us a few days to review your paper and let you know the next and final steps.

Lastly, if your institution or institutions have a press office, please let them know about your upcoming paper now to help maximize its impact. If they'll be preparing press materials, please inform our press team within the next 48 hours. Your manuscript will remain under strict press embargo until 2 pm Eastern Time on the date of publication. For more information, please contact onepress@plos.org.

You will receive an invoice from PLOS for your publication fee after your manuscript has reached the completed accept phase. If you receive an email requesting payment before acceptance or for any other service, this may be a phishing scheme. Learn how to identify phishing emails and protect your accounts at https://explore.plos.org/phishing.

If we can help with anything else, please email us at customercare@plos.org.

Thank you for submitting your work to PLOS ONE and supporting open access.

Kind regards,

PLOS ONE Editorial Office Staff

on behalf of

Dr. Balbir B. Singh

Academic Editor

PLOS One
